# Fabrication of Stretchable Piezoelectric Sensor with a Kirigami Design for Heart Sound Monitoring

**DOI:** 10.3390/s25237253

**Published:** 2025-11-28

**Authors:** Xudong Zhang, Xudong Ye, Xi Lei, Hong Hu, Hai Liu, Shaobo Jin, Guoyong Ye, Tingting Zhao

**Affiliations:** 1 Key Laboratory of Advanced Display and System Applications, Ministry of Education, Shanghai University, Shanghai 200072, China; 2 The School of Microelectronics, Shanghai University, Shanghai 201800, China; 3 Henan Provincial Key Laboratory of Intelligent Manufacturing of High-End Equipment, Zhengzhou University of Light Industry, Zhengzhou 450002, China; 4 Shanghai Key Laboratory of Intelligent Manufacturing and Robotics, Shanghai University, Shanghai 200444, China

**Keywords:** flexible piezoelectric sensor, PVDF, Kirigami structure, heart sound monitoring, stretchable device

## Abstract

Heart sounds contain critical information about valve activity and hemodynamics, serving as an essential basis for cardiovascular disease diagnosis. However, traditional heart sound sensors are either rigid or flexible but non-stretchable, limiting their ability to accommodate chest deformation and leading to signal distortion. This study proposes an easy-to-fabricate, stretchable piezoelectric heart sound sensor with a Kirigami-inspired design, a five-layer “sandwich” structure. Periodic Kirigami cuts significantly enhance stretchability while maintaining piezoelectric conversion efficiency. Finite element simulations reveal the Kirigami structure is more sensitive to hinge length and thickness than to hinge width. Electrical tests demonstrate a linear response to sound pressure, with output voltage rising from 0.11 V to 0.42 V (70–94 dB), and voltage amplitude increasing from 9 mV to 0.35 V (60–160 Hz). The sensor exhibits excellent stability, with a maximum amplitude variation of approximately 11% under 0–30% strain, a 17% voltage decrease at 11 mm bending radius, and less than 9% output fluctuation during 1200 s continuous excitation. Seven-day monitoring confirms reliable detection of the first (S1) and second (S2) heart sounds, with signals highly consistent with ECG and a commercial sensor, verifying its potential for wearable long-term monitoring and early cardiovascular disease screening.

## 1. Introduction

As direct acoustic representations of the physiological activities of the cardiovascular system, heart sound signals contain key pathological information (e.g., heart valve opening/closing and hemodynamics) and serve as an important basis for the early diagnosis of diseases such as coronary heart disease and heart failure [1,2,3,4]. Traditional stethoscopes rely on the experience of physicians, suffering from limitations including strong subjectivity and difficulty in continuous monitoring [5,6]. Existing electronic auscultation devices are mostly based on rigid sensors, which are hard to deploy portably due to their large size. Furthermore, the rigid structure has poor adherence to the skin and cannot adapt to skin deformation during human movement, failing to meet the requirements of wearable long-term continuous monitoring [7,8,9]. The development of flexible sensors provides a solution to this problem—their flexibility enhances adherence to the skin, enabling adaptation to long-term monitoring scenarios.

Piezoelectric polymers (e.g., Polyvinylidene Fluoride (PVDF)) have become one of the core materials for flexible sensors owing to their excellent flexibility and biocompatibility, and they exhibit unique advantages especially in cardiovascular signal monitoring [10,11,12]. Their thin-film form can closely adhere to the skin, realizing high-fidelity capture of weak physiological vibrations such as heart activity and pulses. For instance, previous studies have used sheet-like PVDF to construct a mattress-type unobstructive measurement system, which simultaneously acquires heart sounds, ballistocardiogram (BCG), and respiratory signals during infant sleep. The obtained pulse interval is highly consistent with the electrocardiogram (ECG) reference value, verifying its reliability in unobstructive vital sign monitoring [13]. Another study integrated flexible PVDF films into wearable chest patches to capture high-fidelity forcecardiography (FCG) and respiratory signals; the extracted heartbeat interval (R^2^ > 0.99) and respiratory interval (R^2^ > 0.96) are highly consistent with the results of clinical-grade devices, demonstrating excellent wearable monitoring potential [14]. However, pure PVDF films have limited stretchability and are prone to output signal distortion due to structural deformation. Therefore, improving the stretchability of PVDF sensors through structural design has become a key step in expanding their application in wearable physiological signal monitoring. 

To address this limitation, this study designs a flexible PVDF piezoelectric sensor based on the Kirigami structure. The device adopts a five-layer “sandwich” structure of Polyimide (PI)-Electrode-PVDF-Electrode-PI, with the PVDF film as the core sensitive layer. Relying on its high sensitivity to weak mechanical vibrations, it achieves efficient transduction of heart sound signals. Meanwhile, periodic Kirigami cutting is introduced to release geometric constraints, significantly improving the sensor’s stretchability and adaptability to dynamic skin deformation while ensuring mechanical stability. Benefiting from this structural optimization, the sensor can maintain stable detection of heart sound signals for an extended period, and is expected to provide a feasible path for the early screening of cardiovascular diseases and wearable physiological signal monitoring.

## 2. Materials and Methods

### 2.1. Fabrication of the Piezoelectric Sensor with a Kirigami Design

**Figure 1:**
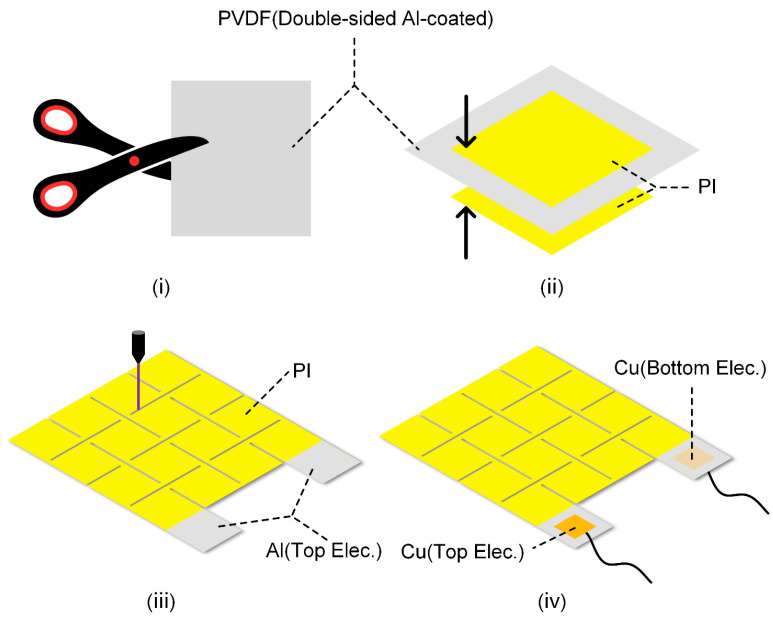
Simplified fabrication process of the sensor.

A double-sided aluminum (Al)-coated PVDF film (30 μm, Jinzhou Kexin Electronic Materials Co., Ltd., Jinzhou (Liaoning Province), China) was used as the piezoelectric sensing layer, which was cut into rectangular pieces (i). Two pieces of single-sided adhesive-backed polyimide films (20 μm, Dongguan Ronghui Insulating Materials Co., Ltd., Dongguan (Guangdong Province), China) were prepared; after peeling off the release films on their surfaces in sequence, they were attached to the upper and lower surfaces of the cut rectangular PVDF film (ii), with parts of the Al electrodes exposed. An ultraviolet nanosecond laser cutting machine (Changzhou Guangqi Laser Technology Co., Ltd., Changzhou (Jiangsu Province), China) was used to engrave the Kirigami structure on the film (iii). Finally, Cu conductive tape was used to attach wires to the exposed Al electrodes (iv), completing the fabrication of the piezoelectric sensor (4 cm × 5 cm). The detailed fabrication process of the piezoelectric sensor was shown in Figure 1.

### 2.2. Finite Element Analysis

The stretchability of different structures was analyzed using the commercial Abaqus 2020. Kirigami patterns with various hinge widths, lengths, and thicknesses were first modeled in AutoCAD 2021 (R.47.0.0), and subsequently imported into Abaqus. All models were assigned the isotropic elastic material property. The materials parameters used in FEA are detailed in Section 3.2. A quasi-static analysis was performed using a dynamic implicit step, in which the kinetic energy was strictly constrained to be no more than 1% of the strain energy and the S4R elements were utilized for mesh generation [15,16]. Displacement loads were then applied to the four corners of the models. The change in stress due to the applied displacements, and changes in area due to the reaction force were numerically calculated, respectively. 

### 2.3. Characterization and Testing

An oscilloscope (TBS2104, Tektronix Technology Co., Ltd., Shanghai, China) was used to measure the electric performance of the sensor. A speaker (3 W, Shenzhen Huayu Feifan Technology Co., Ltd., Shenzhen (Guangdong Province), China) was employed to apply a sound pressure excitation, and a sound level meter (AR844, Smart Sensor Instrument Co., Ltd., Dongguan (Guangdong Province), China) was used to record sound intensity. The piezoelectric sensor and a commercial heart sound sensor (HKY-06E, Hefei Huake Electronic Technology Institute, Hefei (Anhui Province), China) were used to simultaneously record heart sound signals. Detailed information regarding the ECG monitoring experiment is as follows: Three Ag/AgCl gel electrodes (Beijing Huarun Kangtai High-Tech Development Research Institute, Beijing, China) were placed on the left chest, right chest, and right abdomen. Electrodes were connected to a dedicated AD8232 ECG acquisition board (Sichiray Technology Co., Ltd., Wuxi (Jiangsu Province), China) via a three-lead cable. Data was transmitted via Bluetooth to a host computer for real-time display and acquisition. The output of the piezoelectric sensor was amplified and filtered by a self-made acquisition board, and then collected by the oscilloscope. The custom acquisition board operates at a sampling frequency of 1 kHz. It employs an active second-order 200 Hz hardware Butterworth low-pass filter to eliminate high-frequency noise. At the software level, an iirnotch function first designs a 50 Hz notch filter to suppress power line interference, followed by a fourth-order Butterworth filter to extract the signal. The respiratory wave is processed through a 1 Hz low-pass filter, while the heart sound signal undergoes a 20 Hz high-pass filter. The group delay of the hardware filters was measured using Texas Instruments Filter Design Tool (1.0), https://www.ti.com/tool/download/ANALOG-FILTER-DESIGNER/1.0, accessed on 17 October 2025: 1.2 ms at 20 Hz and 1.1 ms at 200 Hz, both significantly shorter than the time interval between S1 and S2 (approximately 260 ms). For software filtering, the filtfilt function was employed to perform zero-phase notch, low-pass, and high-pass filtering, compensating for phase delay. The acquisition board employs a non-inverting proportional voltage amplifier to amplify the signal with a gain of 15. An additional 1.65 V bias voltage is applied to ensure the signal falls within the ADC’s sampling voltage range. The input stage utilizes a charge amplifier, featuring a CMOS precision operational amplifier with an input impedance exceeding 10 TΩ. This design effectively minimizes charge leakage and reduces signal distortion.

## 3. Results and Discussion

### 3.1. Structure and Working Mechanism of the Piezoelectric Sensor

**Figure 2:**
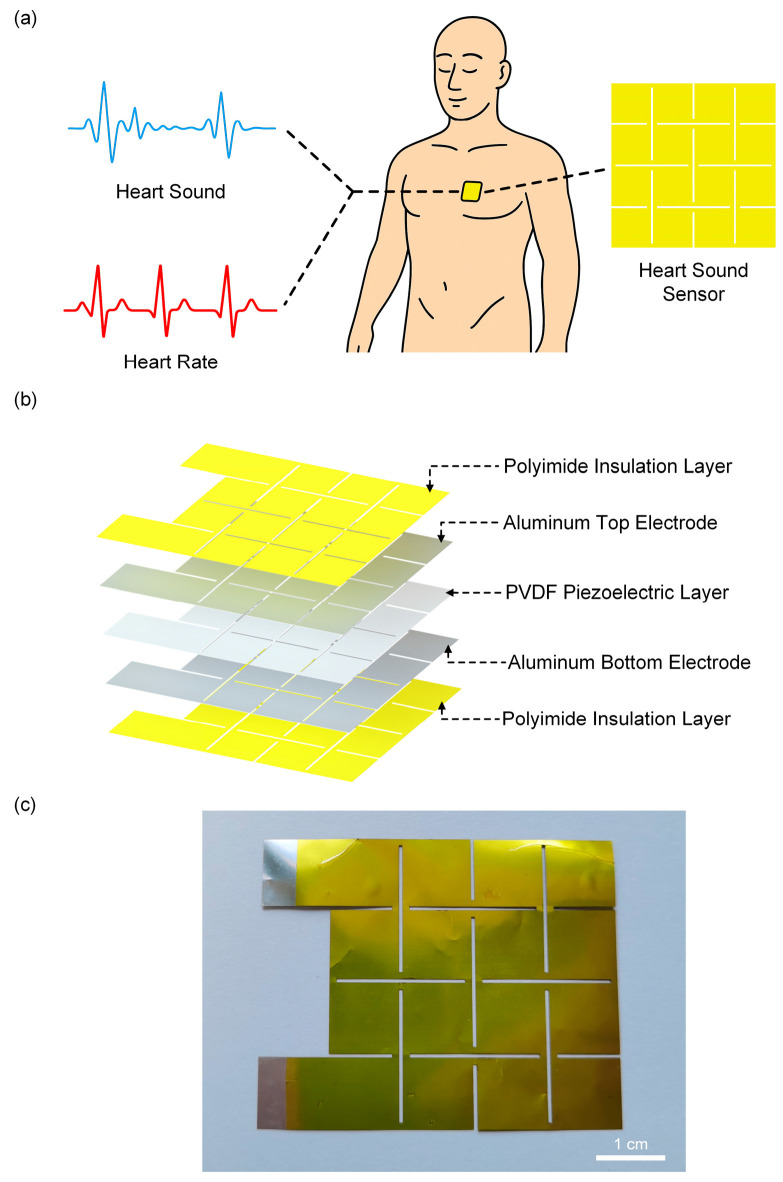
Structure of the piezoelectric sensor. (**a**) The Kirigami-structured sensor attached to the chest for human signal monitoring. (**b**) Exploded view of the piezoelectric sensor structure. (**c**) Photograph of the sensor.

Figure 2a illustrates the piezoelectric sensor placed on the chest for human signal monitoring. The sensor adopts a “sandwich” structure with a Kirigami design, where the top and bottom layers are protective layers made of PI films, and the middle layer is the piezoelectric sensitive layer (Figure 2b). Figure 2c shows a photograph of the sensor, where the silver-colored region corresponds to the exposed Al electrode to facilitate subsequent wire connection.

**Figure 3:**
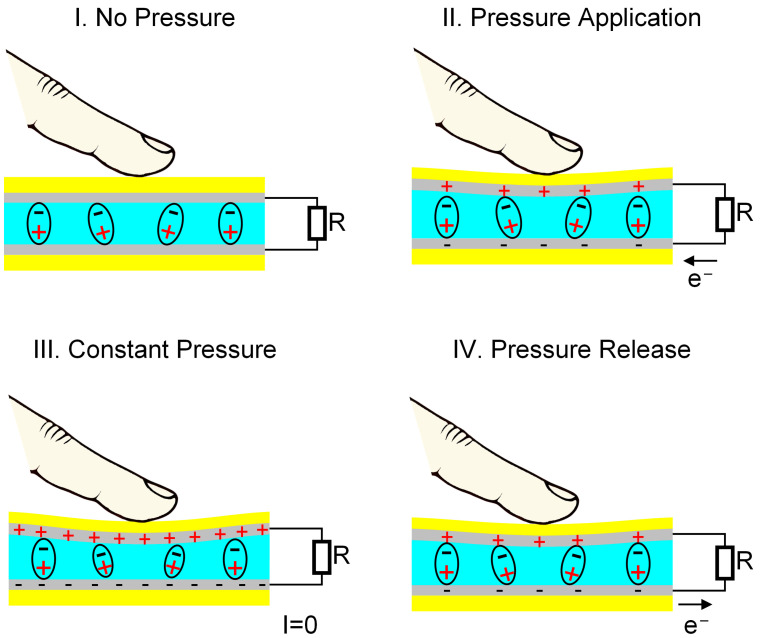
Working mechanism of the sensor. In the figure, the yellow regions are Polyimide (PI), the silver-gray regions are Al electrodes, and the blue regions are PVDF.

The piezoelectric effect of PVDF originates from changes in its internal dipole moment under external force [17,18,19]. Initially, without external force (Figure 3(I)), the net polarization of PVDF film remains stable, free charges on the electrodes are in equilibrium, and no output signal is generated. When under pressure (Figure 3(II)), the internal dipoles shift, increasing net polarization and creating a potential difference between the electrodes, which generates a transient reverse current. Once the applied force no longer changes (Figure 3(III)), the net polarization reaches a steady state, and a new equilibrium is established with no current flow. Upon releasing the force (Figure 3(IV)), the internal dipoles gradually return to their initial state, reducing net polarization. To maintain charge balance, electrons flow from the lower to the upper electrode, producing a forward current. This completes a full pressure-electricity conversion cycle. PVDF offers unique advantages over other piezoelectric materials, such as Pb(Zr, Ti)O_3_, BaTiO_3_, and KNbO_3_, due to its flexibility, thinness, and suitability for wearable designs. Furthermore, its wide frequency response range aligns well with the frequency range of heart sounds (20–200 Hz), making it particularly effective for heart sound detection and monitoring. 

**Figure 4:**
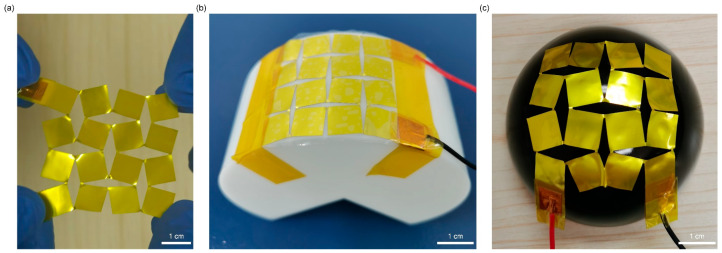
Deformability of the sensor: (**a**) Stretched state; (**b**) Bent state on a cylindrical surface; (**c**) Bent state on a spherical surface.

PVDF films are inherently flexible but lack stretchability. To address this limitation, a Kirigami structure was introduced into the PVDF film. By applying regular cutting patterns, the geometric constraints of the continuous PVDF film are released, endowing the overall structure with higher deformation freedom under stress and thus significantly improving stretchability. Combined with the natural flexibility of PVDF, the Kirigami design enables the device to achieve both excellent flexibility and stretchability, allowing it to conform to the complex curved surfaces (e.g., cylindrical and spherical surfaces as shown in Figure 4). This makes the device adapt well to human skin surface deformation. In addition, the Kirigami structure concentrates tensile stress in the hinge region, while the PVDF sensitive layer is mainly distributed in the sheet region. This design minimizes the impact of tensile deformation on pressure detection performance, achieving “deformation insensitivity” and ensuring stable signal output even under deformation.

### 3.2. Fabrication and Mechanical Properties of the Piezoelectric Sensor with a Kirigami Structure

**Figure 5:**
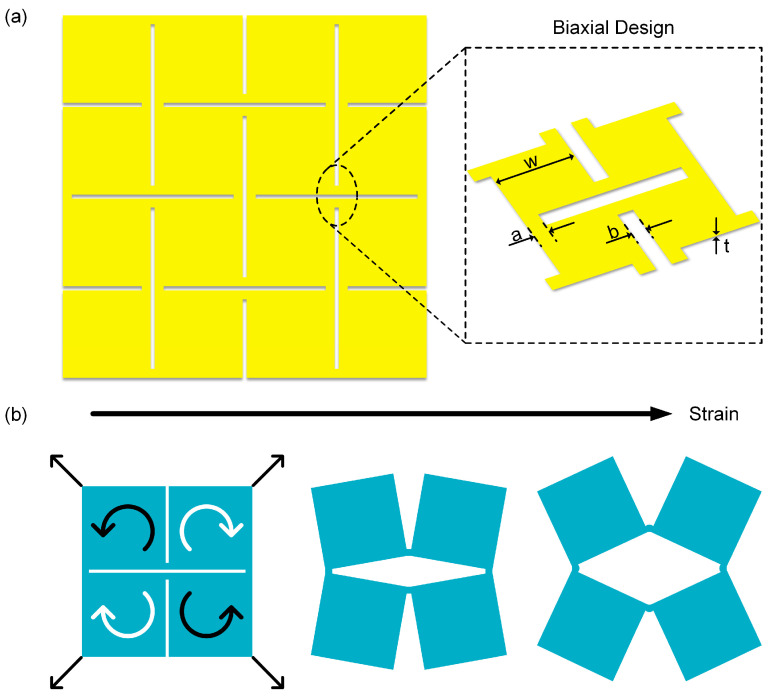
Geometric parameters and mechanical properties of the piezoelectric sensor. (**a**) Schematic diagram of the Kirigami structure with biaxial design on the sensor (the inset shows a magnified view of the structure). (**b**) Schematic diagram of the sensor under tensile deformation.

The Kirigami structure, a geometric design strategy derived from traditional paper-cutting art, endows materials with additional deformation freedom by introducing periodic cuts in continuous films [20,21,22]. This structure allows the material to undergo controlled local rotation and expansion under external force, thereby achieving macroscale stretchability [23,24,25]. As shown in Figure 5a, this structure adopts a biaxial design, and its basic unit is defined by geometric parameters including hinge length a, hinge width b, thickness t, and square unit length w—these parameters directly determine the deformation mode of the cuts. Benefiting from this geometric configuration, the Kirigami unit can redistribute stress during in-plane stretching, significantly improving the stretchability and conformability of the overall film. Figure 5b illustrates the stretching principle of the basic unit in the Kirigami structure. When external tensile force acts on the four corners of the structure, four adjacent square sheets rotate synergistically via the hinges, causing the originally closely arranged sheets to gradually open and form a rhombic gap in the middle. This structure transfers the overall deformation of the film to the hinge region, while the square sheet region is barely affected by stress [26,27,28]. 

Figure 1 illustrates the simplified fabrication process of the piezoelectric sensor with a Kirigami design, which is divided into four key sub-steps (i)–(iv). The piezoelectric sensing layer is composed of a PVDF piezoelectric film and Al electrodes on its upper and lower surfaces. After assembling the protective layer and the piezoelectric sensing layer into an integrated structure, an ultraviolet nanosecond laser cutting machine is used to engrave crisscross “Kirigami” structure on the composite film, finally yielding the desired piezoelectric sensor (for detailed fabrication procedures, see Section 2.1).

**Figure 6:**
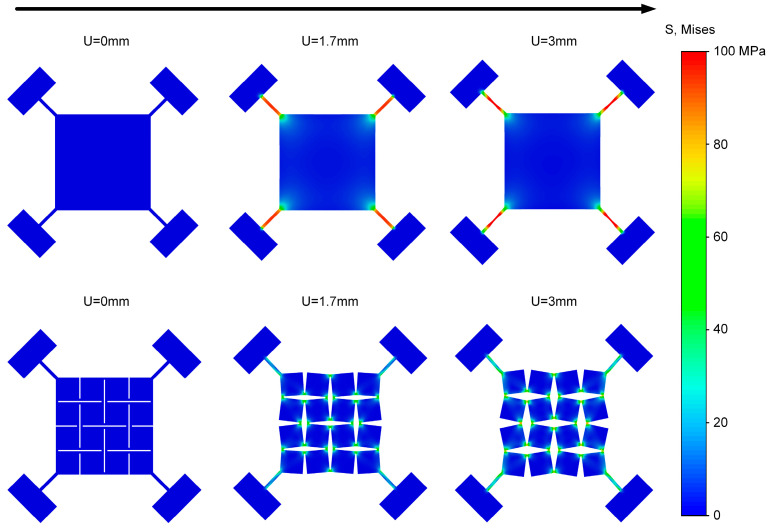
Tensile simulation results of the planar structure and the Kirigami structure.

To compare the load-bearing differences between the Kirigami structure and the planar structure, finite element simulations were conducted (Figure 6). We modeled the PI/PVDF/PI laminate as a single equivalent, homogeneous, isotropic shell that reproduces its in-plane and bending behavior within the small-strain, small-deflection regime. The equivalent material parameters, including Young’s modulus, density, and Poisson’s ratio of PI/PVDF/PI film, were identified by energy equivalence, as presented in Table 1 [29,30]. The thickness of the shell was 70 μm. Simulation results show that under external displacement load, the Kirigami structure effectively alleviates stress concentration and exhibits a better overall mechanical response than the traditional planar structure. For the intact planar structure, as the displacement U increases, stress concentrates rapidly in the four corner regions, exhibiting an obvious stress concentration effect. This not only limits its overall stretchability but also tends to cause premature failure of local materials. In contrast, the Kirigami structure incorporates periodic cuts, which converts external stretching into unit rotation and relative displacement, enabling stress redistribution within the film. As the displacement increases from 0 mm to 3 mm, the basic panel units gradually open up, forming uniform rhombic gaps. It can be observed that stress is mainly concentrated in the hinge regions, while the square panel regions only experience minimal stress—this effectively prevents the core sensing layer from failing, demonstrating the deformation insensitivity characteristic of the Kirigami structure. These results indicate that, based on its periodic cuts, the Kirigami structure outperforms the traditional planar structure in terms of stress concentration, making it a preferred option for stretchable structural designs involving core sensing elements. 

**Figure 7:**
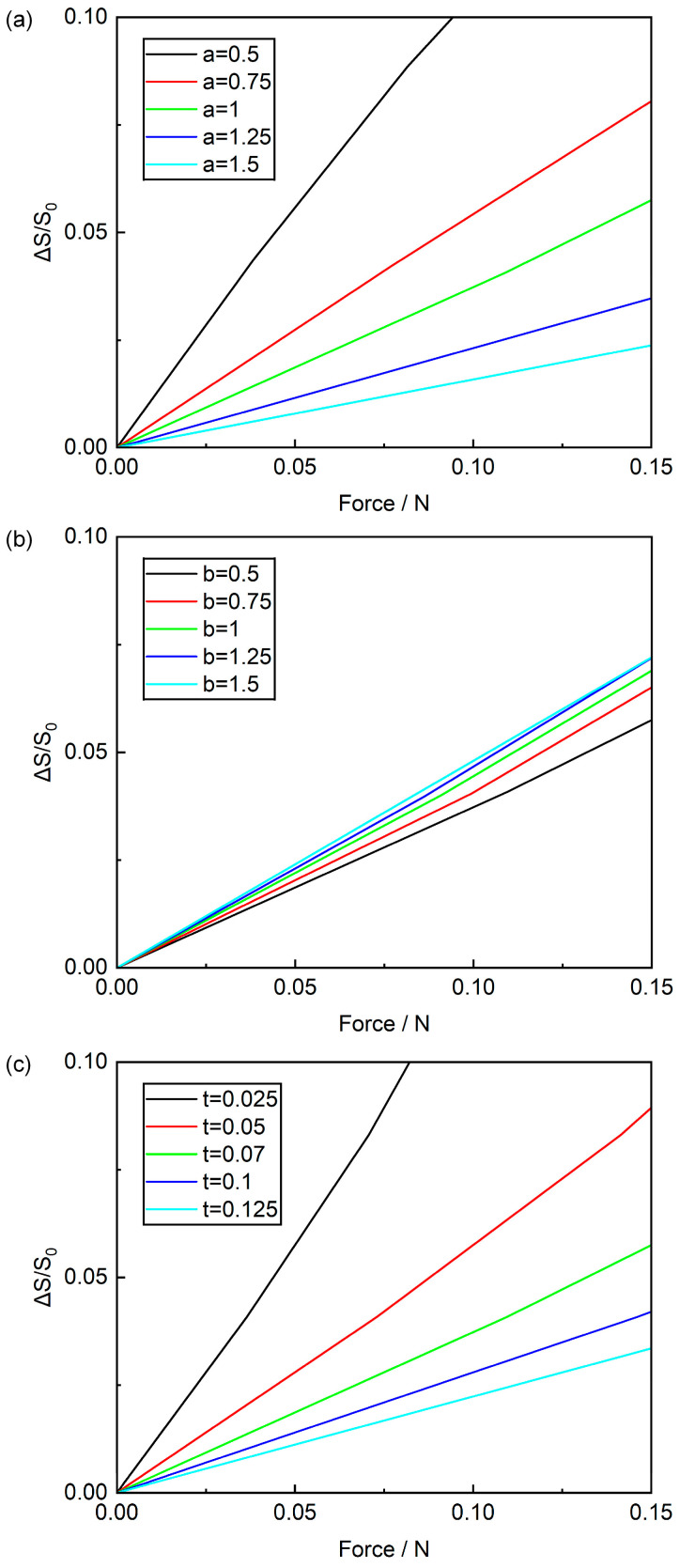
Effects of (**a**) hinge length a, (**b**) hinge width b, and (**c**) hinge thickness t on the area change rate (ΔS/S_0_).

To further clarify the influence of geometric parameters on structural stretchability, stretching simulations were performed by adjusting the hinge length a, hinge width b, and thickness t separately. Tensile reaction forces at each state were extracted using Abaqus tools, where the horizontal axis represents reaction forces at the four corners and the vertical axis denotes the rate of area change (calculated by dividing the area difference resulting from membrane deformation by the original area). Figure 7a plots the relationship between area change rate and tensile reaction force across varying hinge lengths a. It shows that for the same applied force, a smaller hinge length a results in a larger area change rate. Notably, under a 0.05 N tensile force, a 1.5 mm hinge length yields a 0.8% area change rate, while a 0.5 mm hinge length produces a 5.6% rate. This indicates that a smaller hinge length a correlates with superior tensile performance. Figure 7b illustrates how area change rate varies with tensile reaction force at different hinge widths b. It reveals that for the same applied force, a larger hinge width b leads to a higher area change rate. Specifically, when subjected to a 0.05 N tensile force, a 0.5 mm hinge width produces a 1.9% area change rate, whereas a 1.5 mm hinge width yields a 2.5% area change rate. This suggests that a larger hinge width b enhances tensile properties, but its impact on area change rate is relatively minor compared to that of hinge length and thickness. Figure 7c displays how the area change rate varies with tensile reaction force for different hinge thicknesses t. It indicates that for the same applied force, a thinner hinge thickness t yields a larger area change rate. In particular, under a 0.05 N tensile force, a 0.125 mm hinge thickness produces a 1.1% area change rate, while a 0.025 mm hinge thickness achieves a 5.8% area change rate. This confirms that a smaller hinge thickness t improves better tensile properties. Integrating these simulation results and analyses, we conclude that to enhance the tensile properties of Kirigami structures, it is advisable to use slender and thin hinges—specifically, a smaller hinge length a, a larger hinge width b, and a thinner hinge thickness t. Additionally, for manufacturing and design convenience, this study selected a hinge length (a) of 1 mm, a hinge width (b) of 0.5 mm, a hinge thickness (t) of 0.07 mm, and a square panel size (w) of 1 cm.

### 3.3. Characterization of the Electrical Properties of the Piezoelectric Sensor

**Figure 8:**
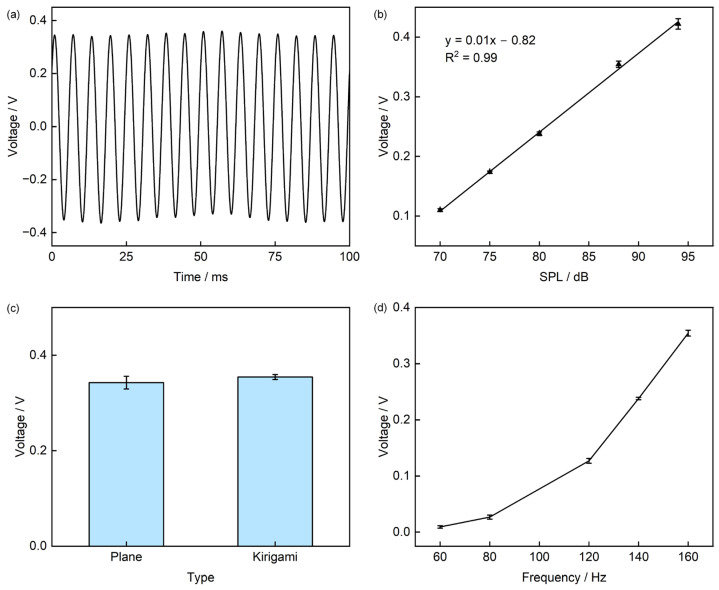
Electrical Performance Characterization of the Sensor. (**a**) Output voltage under sound pressure excitation at 160 Hz and 88 dB; (**b**) Effect of different Sound Pressure Levels (SPL) on output voltage; (**c**) Comparison of output voltage before and after cutting; (**d**) Frequency response of the sensor in the range of 60–160 Hz.

To facilitate the testing of heart sound signals, a loudspeaker excitation method is adopted (The specific test apparatus is shown in Figure S1. When the piezoelectric sensor is subjected to a sound pressure of 88 dB at a frequency of 160 Hz, its output voltage is approximately 0.35 V (Figure 8a). The sensitivity curve of the sensor is plotted in Figure S2. To investigate the effect of Sound Pressure Level (SPL) on the output characteristics, we kept the frequency constant and varied the SPL; the results show that the output voltage exhibits an approximately linear increase with increasing SPL, with an R^2^ value of 0.99. When the SPL increases from 70 dB to 94 dB, the output voltage rises from 0.11 V to 0.42 V (Figure 8b). Considering that cutting the sensor into a Kirigami structure might affect its piezoelectric performance, we conducted sound pressure excitation tests on both the planar-structured and Kirigami-structured sensors to verify whether the structural optimization impairs the piezoelectric conversion efficiency of PVDF. Under sound pressure excitation at 88 dB and 160 Hz, the output voltages of the two structures are 0.34 V and 0.35 V, respectively, with a relative difference of only 2.9% (Figure 8c). This result confirms that Kirigami cutting only alters the sensor’s structural mechanical properties and does not cause adverse effects on its piezoelectric conversion efficiency. With the SPL fixed, we further tested the effect of excitation frequency (60 Hz to 160 Hz) on the output, obtaining a frequency response curve: as the frequency increases from 60 Hz to 160 Hz, the output voltage rises from 9 mV to 0.35 V (Figure 8d). We also characterized the Signal-to-Noise Ratio (SNR) in Figure S3, which showed that the sensor had a SNR of 10 dB at 60 Hz and 42 dB at 160 Hz, indicating good signal quality.

**Figure 9:**
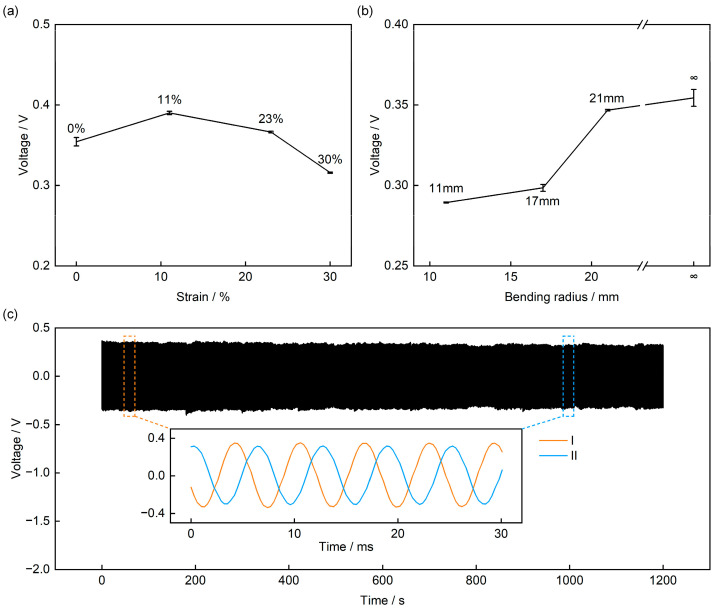
Electrical Performance of the Sensor under Mechanical Deformation and Long-Term Stability Tests. (**a**) Output voltage under tensile conditions. (**b**) Output voltage under bending conditions. (**c**) 1200 s cyclic stability test.

Notably, owing to the introduction of the Kirigami structure, the sensor exhibits the key characteristic of insensitivity of the output response to deformation—we applied four levels of tensile deformation (0%, 11%, 23%, and 30%) to four sensors, and their output voltage only showed minor fluctuations (Figure 9a and Figure S4), with a maximum variation of approximately 11% (compared to the 0% tensile state). This is because when the sensor deforms, the square panels undergo almost only in-plane rotation and displacement, without causing additional stress damage to the PVDF sensing layer. To meet the practical application requirement of heart sound signal measurement on the human skin surface, we further characterized the sensor’s output performance under different bending conditions (Figure 9b). Under the same SPL and frequency, the sensor’s output decreases slightly as the bending radius decreases: when the bending radius is 11 mm, the output voltage is 0.29 V, a decrease of approximately 17% compared to the non-bent state. The reason for this may be that as the bending radius decreases, some regions of the square panels become nearly perpendicular to the sound pressure plane, leading to reduced sound pressure reception efficiency; nevertheless, the sensor still maintains a relatively high output amplitude, demonstrating its stable operating capability on curved surface. 

Finally, to verify the long-term reliability of the sensor, we performed a 1200 s continuous sound pressure excitation test on it (88 dB, 160 Hz). The results show that the sensor’s output voltage remains essentially stable, with a fluctuation range of less than 9% (Figure 9c). Magnifying the voltage waveforms around the two time points (60 s [I] and 1000 s [II]) reveals that the output voltage decreases from the initial 0.35 V to 0.32 V. This slight decrease may stem from minor sound pressure drift caused by the long-term operation of the loudspeaker. Nonetheless, the sensor’s output still remains at a relatively high voltage level (approximately 0.32 V), fully confirming its long-term operational stability.

### 3.4. Application in Heart Sound Monitoring

**Figure 10:**
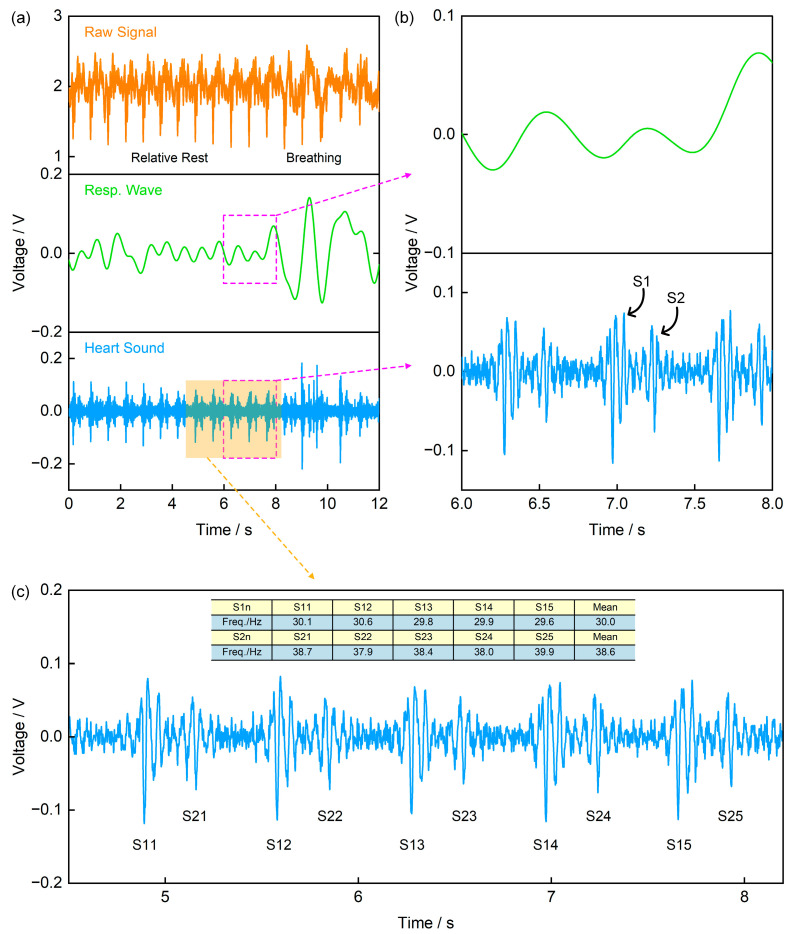
Application of the Piezoelectric Sensor in Heart Sound Monitoring. (**a**) The recorded original signal, along with the respiratory wave and heart sound signal obtained via a 1 Hz low-pass filter and a 20–200 Hz band-pass filter, respectively; (**b**) Enlarged view of the signal in the 6–8 s time window from (**a**), where the upper trace is the respiratory wave and the lower trace is the heart sound signal; (**c**) Average frequencies of the first heart sound (S1) and the second heart sound (S2).

Heart sound signals are acoustic manifestations of mechanical vibrations generated during cardiac contraction and relaxation, and are widely used in disease diagnosis [1,2,3,4]. In a normal cardiac cycle, the main components are the first heart sound (S1, generated by atrioventricular valves closure) and the second heart sound (S2, generated by semilunar valves closure) [4,31,32]. Figure 10a presents the original signal and its processing results recorded by the self-made piezoelectric signal acquisition board with a sampling frequency of 1000 Hz. Specifically, the original signal was processed using a 1 Hz low-pass filter and a 20–200 Hz band-pass filter to obtain the respiratory wave and heart sound signal, respectively. Figure 10b is an enlarged view of the signal in the 6–8 s time window in Figure 10a, from which the S1 and S2 components of the heart sound signal can be clearly identified. Furthermore, by expanding the signal analysis range to 5 consecutive cardiac cycles and calculating the frequencies of the two components separately, the results show that the dominant frequency of the S2 component (mean: 38.6 Hz) is slightly higher than that of S1 (mean: 30 Hz) (Figure 10c). This frequency difference aligns with the physiological mechanism of heart sound generation—semilunar valves close faster than atrioventricular valves, leading to higher-frequency mechanical vibrations, thus demonstrating the typical characteristics of normal heart sound signals.

**Figure 11:**
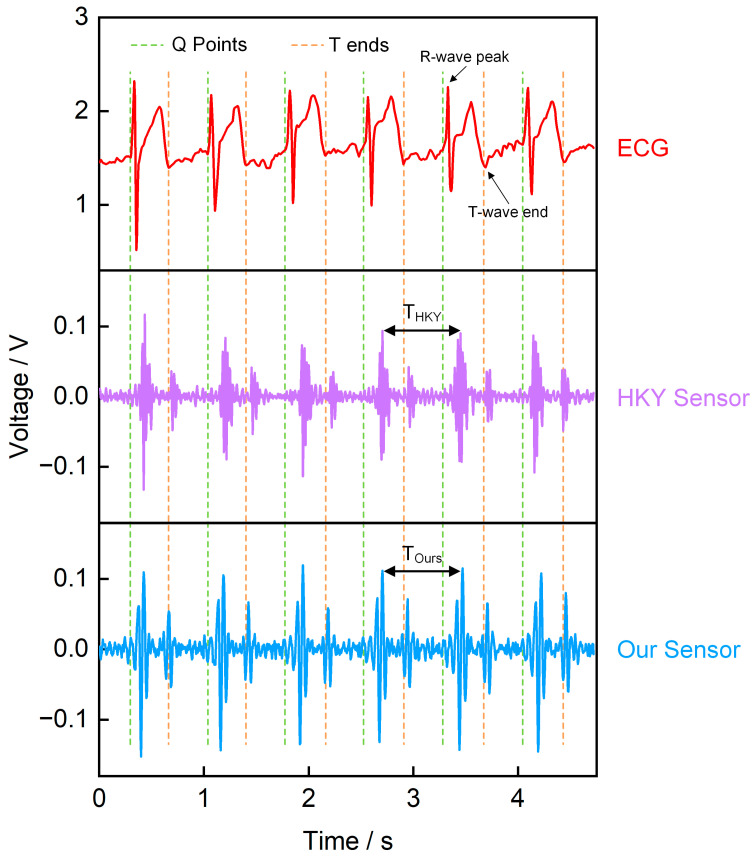
Simultaneously recorded waveforms. The upper trace is the ECG, the middle trace is the commercial sensor signal, and the lower trace is our piezoelectric sensor signal.

To verify the reliability of the piezoelectric sensor in monitoring heart sound signals, we used an ECG sensor, our piezoelectric sensor, and a commercial heart sound sensor (HKY sensor) to record heart sounds, simultaneously (Figure 11). The results indicate a clear temporal correlation between the ECG signal and the heart sound signal. Specifically, the QRS complex in the ECG marks the start of ventricular contraction, immediately followed by S1, which is generated by the closure of the atrioventricular valves; in contrast, S2, which appears near the T wave, corresponds to the closure of the semilunar valves and the onset of ventricular diastole [4,33,34,35]. We compared the heart sound cycles recorded by our sensor and commercial sensor, both measuring the S1–S1 peak time interval (Table S1). Results showed that the Mean Absolute Error (MAE) between our sensor and commercial sensor was 0.015 s, with a Mean Relative Error (MRE) of 2.08%. These small errors indicate that our sensor effectively records heart sound signals. Additionally, we compared the temporal correlation between our sensor signals and ECG signals. Figure S5a shows the correspondence between the R-wave peak time of the ECG and the S1 peak time of our sensor, while Figure S5b illustrates the correspondence between the T-wave end time of the ECG and the S2 peak time of our sensor. The fitting results from both figures demonstrate that the heart sound signals measured by our sensor exhibit well temporal correspondence with the ECG signals. In summary, comparisons with ECG and commercial sensors confirm the reliability of our piezoelectric sensor in heart sound monitoring.

**Figure 12:**
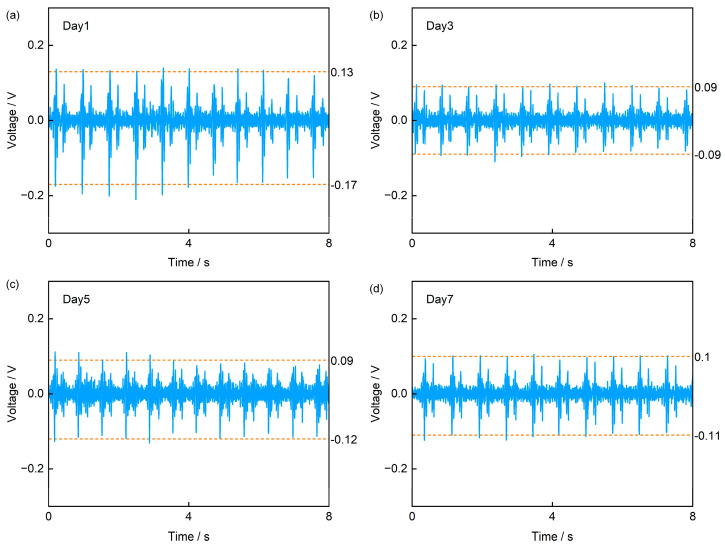
Comparison of heart sound signals from periodic tests over 7 days. (**a**) Day 1, (**b**) Day 3, (**c**) Day 5, (**d**) Day 7.

Finally, to verify the sensor’s feasibility for long-term monitoring, we conducted a test in which the sensor was worn on the left precordial area over a 7-day period using 3M medical transparent adhesive tape, with measurements taken every two days (Figure 12). The acquired signals clearly show the characteristics of S1 and S2 peaks of heart sound signals. Further analysis across the four tests reveals that the sensor’s output voltage reached approximately 0.13 V in the first test, slightly higher than the 0.09 V and 0.1 V observed in subsequent tests. We attribute this discrepancy to that the adhesive tape was initially stronger; after prolonged wear, its adhesiveness weakened, leading to partial detachment at the skin–device interface. This reduced contact explains the lower signal amplitudes in later measurements. Notably, while the amplitude decreased, the signal frequency content remained unchanged. Based on test results and analysis, our sensor demonstrates potential for application in long-term heart sound monitoring scenarios.

## 4. Conclusions

This study proposes and verifies a flexible PVDF piezoelectric heart sound sensor based on Kirigami structure. The device adopts a five-layer “sandwich” structure of PI-Electrode-PVDF-Electrode-PI. While maintaining flexibility, it releases geometric constraints via the Kirigami structure, achieving high adaptability to dynamic skin deformation. Finite element simulations and geometric parameter tuning further demonstrate that the Kirigami design facilitates effective stress transfer. Specifically, stretchability exhibits higher sensitivity to hinge length a and thickness t, but relatively low sensitivity to hinge width b—thereby providing guidance for subsequent designs. In terms of electrical characterization, the sensor exhibits an approximately linear output in response to sound pressure excitation. When the SPL increases from 70 to 94 dB, the output voltage rises from approximately 0.11 V to 0.42 V; when the frequency is scanned from 60 to 160 Hz, the output voltage increases from roughly 9 mV to 0.35 V. Under stretching and bending conditions, the device maintains stable output performance with “deformation insensitivity”: at tensile levels of 0%, 11%, 23%, and 30%, the maximum amplitude variation is approximately 11%; When the bending radius is reduced to 11 mm, the voltage is about 0.29 V, which is a decrease of roughly 17% compared to the planar state—nevertheless, it still maintains a favorable voltage level. Long-term and cyclic tests also confirm its stability: under 1200 s of continuous excitation, the output fluctuation is within approximately 9%; in the 7-day heart sound measurements with the sensor attached to the chest, the S1 and S2 components can still be clearly identified. Additionally, the synchronized comparison with the ECG sensor and commercial heart sound sensor reveals a clear temporal correlation. Overall, the Kirigami-structured PVDF piezoelectric sensor developed in this study achieves a balance among flexibility, stretchability, and output stability. It can reliably capture heart sound signals under stretching, bending, and long-term wear conditions, providing a feasible approach for wearable long-term heart sound monitoring and early screening of cardiovascular diseases. It is worth noting that the sample size n for human subjects in this study is only 1, which may result in the limitations in terms of sample representativeness and population universality. We plan to expand the sample size and integrate clinical needs in the future to further enhance the sensor’s application value.

## Figures and Tables

**Table 1:** Comparison of physical parameters of PI, PVDF, and PI/PVDF/PI film.

	PI	PVDF	PI/PVDF/PI
Young’s modulus (MPa)	2500	2500	2500
Poisson’s ratio	0.34	0.35	0.34
Density (kg/m^3^)	1.40 × 10^12^	1.78 × 10^12^	1.40 × 10^12^
Thickness (μm)	20	30	70

## Data Availability

The data presented in this study are available on request from the corresponding author.

## Supplementary Materials

The following supporting information can be downloaded at: https://www.mdpi.com/article/10.3390/s25237253/s1, Figure S1: Sound Pressure Excitation Test Diagram, Figure S2: Sensor Sensitivity (mV/Pa) Curve, Figure S3: Signal-to-noise ratio (SNR) of the sensor, Figure S4: Output voltages of other three identical sensors (Figure a, Figure b, Figure c) under different tensile conditions, Figure S5: Sensor Temporal Correlation; Table S1: Average Time Difference in Heart Sound Cycles Recorded by Our Sensor and Commercial Sensor.

## Abbreviations

The following abbreviations are used in this manuscript:

PVDF	Polyvinylidene Fluoride
ECG	electrocardiogram
BCG	ballistocardiogram
FCG	forcecardiography
PI	Polyimide
Al	aluminum
SPL	Sound Pressure Level
S1	the first heart sound
S2	the second heart sound
MAE	Mean Absolute Error
MRE	Mean Relative Error
SNR	Signal-to-Noise Ratio
