# Supplementary material for: Fabrication of Stretchable Piezoelectric Sensor with a Kirigami Design for Heart Sound Monitoring
Source: Sensors (Basel). 2025 Nov 28;25(23):7253. doi: 10.3390/s25237253 (PMC12694543; doi:10.3390/s25237253)
Supplement: Supplementary file 1 [file sensors-25-07253-s001.zip › sensors-3968003-supplementary.pdf]

sensors

MDPI

Fabrication of Stretchable Piezoelectric Sensor with a Kirigami Design for Heart Sound Monitoring

Xudong Zhang [1], Xudong Ye [1], Xi Lei [2], Hong Hu [2], Hai Liu [1], Shaobo Jin [3], Guoyong Ye [3], and Tingting Zhao [1,4*]

[1] Key Laboratory of Advanced Display and System Applications, Ministry of Education, Shanghai University, Shanghai 200072, China; 1246093350@shu.edu.cn (X.Z.); 17871621638@shu.edu.cn (X.Y.); hliu5@shu.edu.cn (H.L.)
[2] The School of Microelectronics, Shanghai University, Shanghai 201800, China; 24723668@shu.edu.cn (X.L.); honghu@shu.edu.cn (H.H.)
[3] Henan Provincial Key Laboratory of Intelligent Manufacturing of High-End Equipment, Zhengzhou University of Light Industry, Zhengzhou 450002, China; shaobo_zzu@163.com (S.J.); guoyongye2021@163.com (G.Y.)
[4] Shanghai Key Laboratory of Intelligent Manufacturing and Robotics, Shanghai University, Shanghai 200444, China
* Correspondence: ttzhao@shu.edu.cn

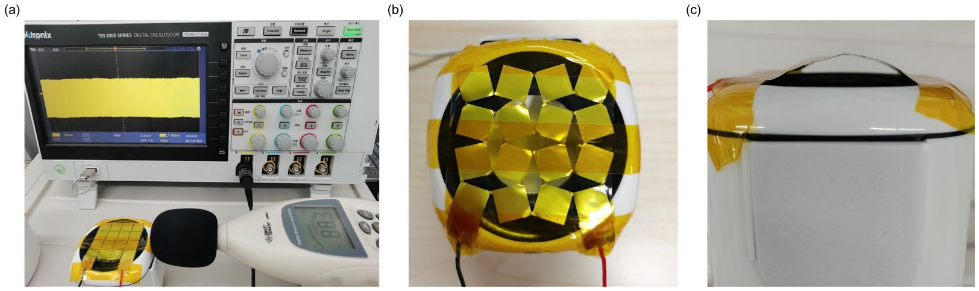

Figure S1. Sound Pressure Excitation Test Diagram. (a) Specific test apparatus. (b) Stretched Film Fixed to the Speaker. (c) Curved Film Fixed to the Speaker.

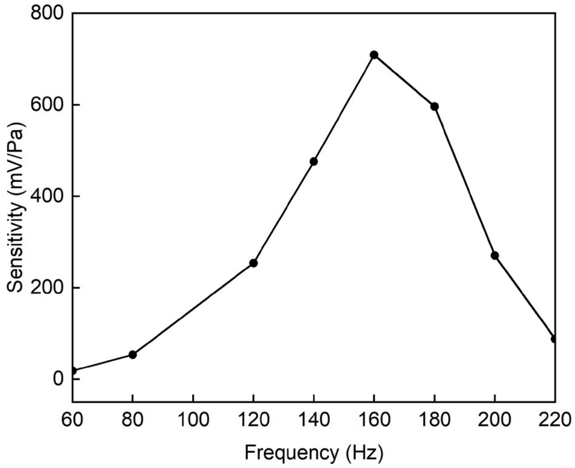

Figure S2. Sensor Sensitivity (mV/Pa) Curve.

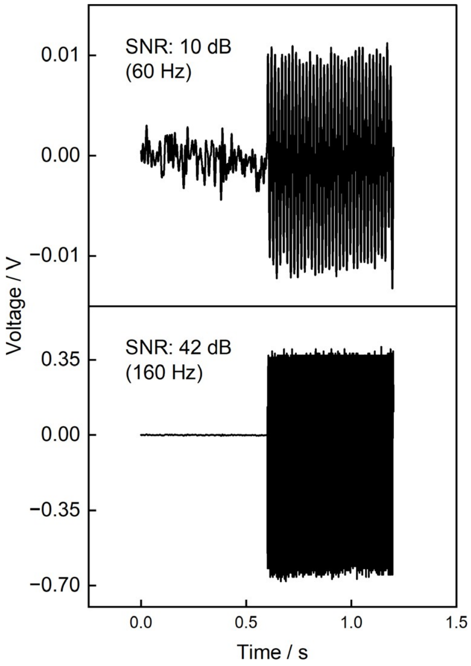

Figure S3. Signal-to-noise ratio (SNR) of the sensor. The SNR at 60Hz is 10dB, and at 160Hz it is 42dB.

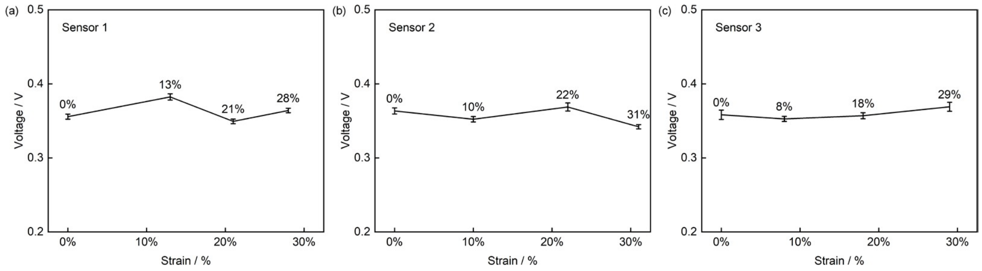

Figure S4. Output voltages of other three identical sensors (Figure a, Figure b, Figure c) under different tensile conditions.

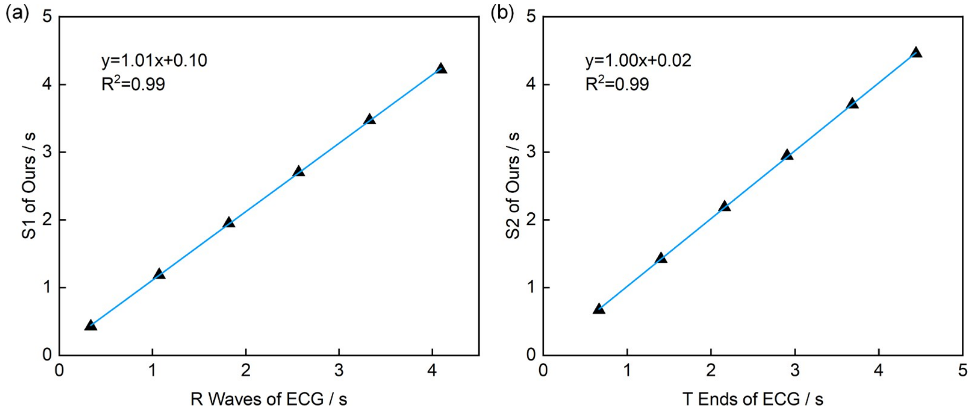

Figure S5. Sensor Temporal Correlation. (a) Temporal Correlation between R-wave Peak and S1 Peak. (b) Temporal Correlation between T-wave End and S2 Peak.

Table S1. Average Time Difference in Heart Sound Cycles Recorded by Our Sensor and Commercial Sensor.

Number	T_{HKY} / s	T_{Ours} / s	AE / s	MAE / s	RE	MRE
1	0.768	0.760	0.008		1.04%	
2	0.756	0.759	0.003		0.40%	
3	0.741	0.755	0.014	0.015	1.89%	2.08%
4	0.741	0.768	0.027		3.64%	
5	0.725	0.750	0.025		3.45%	
